# Necrotizing fasciitis of the head and neck – clinical features, diagnostics, and management strategies

**DOI:** 10.18632/oncoscience.639

**Published:** 2025-12-23

**Authors:** Anna Aydin, Lawik Revend, Doha Revend, Oliver Schuck, Florian Dudde

**Affiliations:** ^1^Department of Oral and Maxillofacial Surgery, Army Hospital Hamburg, Hamburg, Germany; ^2^Department of Plastic Surgery, Army Hospital Berlin, Berlin, Germany; ^3^Department of Otolaryngology, Head and Neck Surgery, Army Hospital Berlin, Berlin, Germany

**Keywords:** necrotizing fasciitis, cervicofacial infection, surgical emergency, debridement, airway management

## Abstract

Background: Necrotizing fasciitis (NF) of the head and neck is a rare but rapidly progressive and life-threatening soft tissue infection that constitutes a true surgical emergency. Due to the complex anatomy of the cervicofacial region and the proximity to the upper airway, early diagnosis and management are particularly challenging, and delayed recognition is associated with high morbidity and mortality. This article aims to provide a concise and clinically oriented overview of the presentation, diagnostic pitfalls, and current management strategies for cervicofacial necrotizing fasciitis.

Methods: A narrative review of the available literature was conducted and complemented by clinical experience from a tertiary referral center. Key aspects including etiology, risk factors, clinical features, imaging findings, laboratory parameters, microbiology, surgical management, airway control, and adjunctive therapies were synthesized and critically discussed.

Results: Cervicofacial NF often presents with disproportionate pain, rapidly progressive swelling, and early systemic toxicity. Odontogenic infections represent the most common source, frequently in the presence of systemic comorbidities such as diabetes mellitus or immunosuppression. Contrast-enhanced computed tomography is the imaging modality of choice, whereas laboratory scoring systems such as the LRINEC score show limited sensitivity in head and neck infections. The cornerstone of treatment is immediate and aggressive surgical debridement combined with broad-spectrum intravenous antibiotics, early airway protection, and intensive care support. Repeated surgical interventions are frequently required. The role of adjunctive hyperbaric oxygen therapy remains controversial and cannot be routinely recommended based on current evidence.

Conclusion: Necrotizing fasciitis of the head and neck requires a high index of suspicion, prompt imaging, and decisive multidisciplinary management. Early surgical intervention and airway control are critical determinants of outcome. Given the rarity of cervicofacial NF, further multicenter studies and registries are needed to refine diagnostic tools, identify prognostic factors, and optimize treatment strategies, particularly in high-risk populations such as immunocompromised and oncologic patients.

## INTRODUCTION

Necrotizing fasciitis (NF) of the head and neck region is a rare but life-threatening soft tissue infection characterized by rapidly progressing fascial necrosis, systemic toxicity, and high mortality [1, 2]. It represents a surgical emergency that requires prompt recognition and aggressive multidisciplinary treatment. The condition most frequently arises in the lower extremities or abdominal wall; cervicofacial manifestations account for fewer than 5% of cases but are associated with unique diagnostic and therapeutic challenges due to the complex anatomy and proximity to the upper airway [3, 4].

While the underlying etiology is often odontogenic, other sources include pharyngeal or parotid infections, trauma, or iatrogenic injury [5–7]. Systemic risk factors such as diabetes mellitus, immunosuppression, alcoholism, and chronic renal failure are frequently present and significantly influence the clinical course and prognosis [8].

## CLINICAL PRESENTATION

Patients with cervicofacial NF often present with severe pain, swelling, erythema, and crepitus, often disproportionate to clinical findings [9]. As the infection progresses, cutaneous manifestations such as skin discoloration, bullae, or necrosis may appear. Systemic signs include fever, tachycardia, hypotension, and signs of sepsis [10]. The progression from cellulitis to deep fascial involvement can occur within hours. Airway compromise is a critical and early concern in head and neck NF [6, 11]. Trismus, dysphagia, and dyspnea can be early indicators of descending infection and possible airway obstruction [4–6, 11]. In many cases, early elective intubation or tracheotomy is essential to secure the airway.

## DIAGNOSTIC STRATEGIES

**Figure 1:**
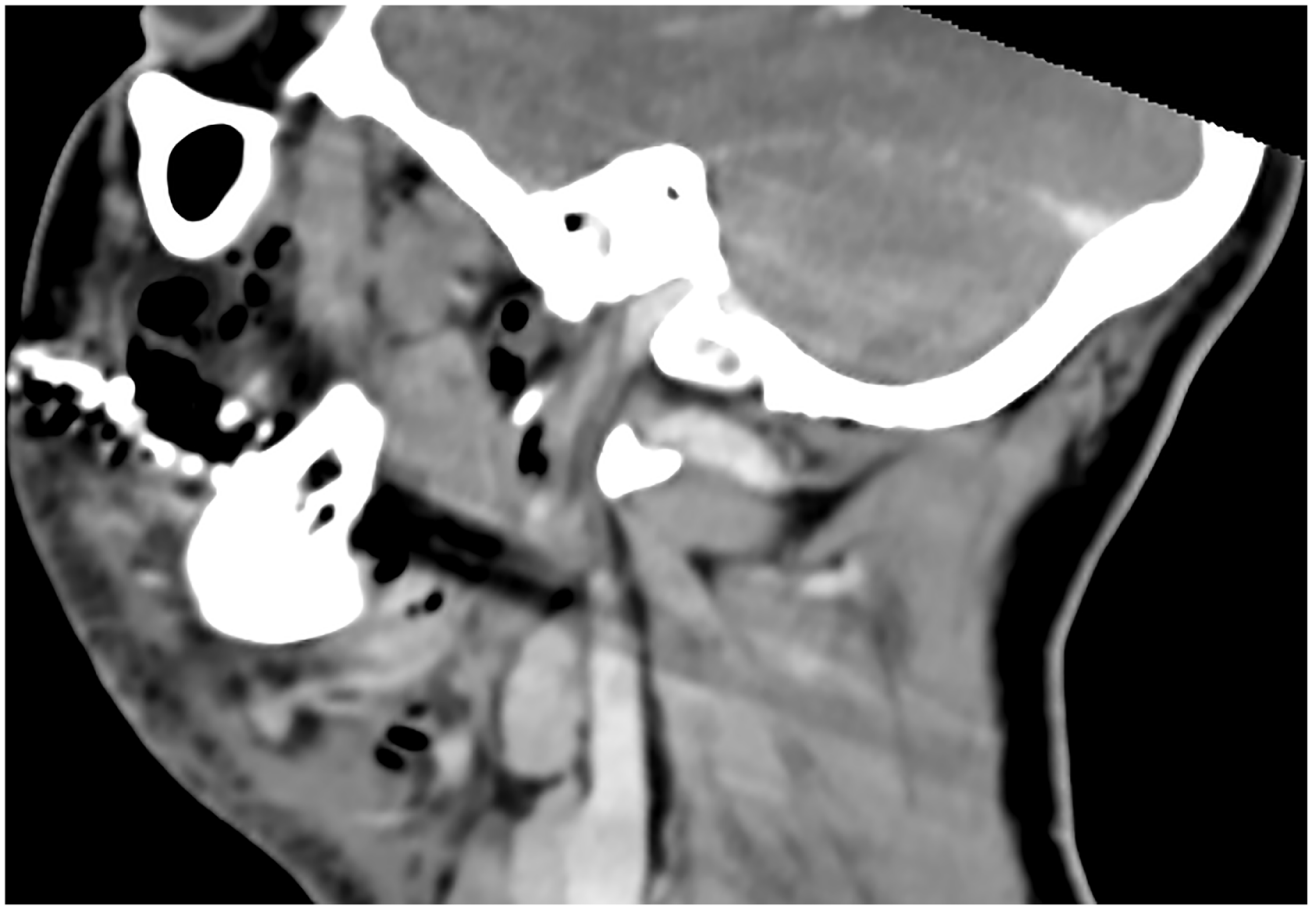
Sagittal CT imaging in cervicofacial necrotizing fasciitis. Sagittal contrast-enhanced computed tomography (CT) scan of the neck demonstrating hallmark features of cervicofacial necrotizing fasciitis, including subcutaneous emphysema, diffuse fascial thickening, and fluid tracking along the deep cervical spaces. The sagittal plane highlights the craniocaudal spread of infection and illustrates the rapid progression along anatomical compartments.

**Figure 2:**
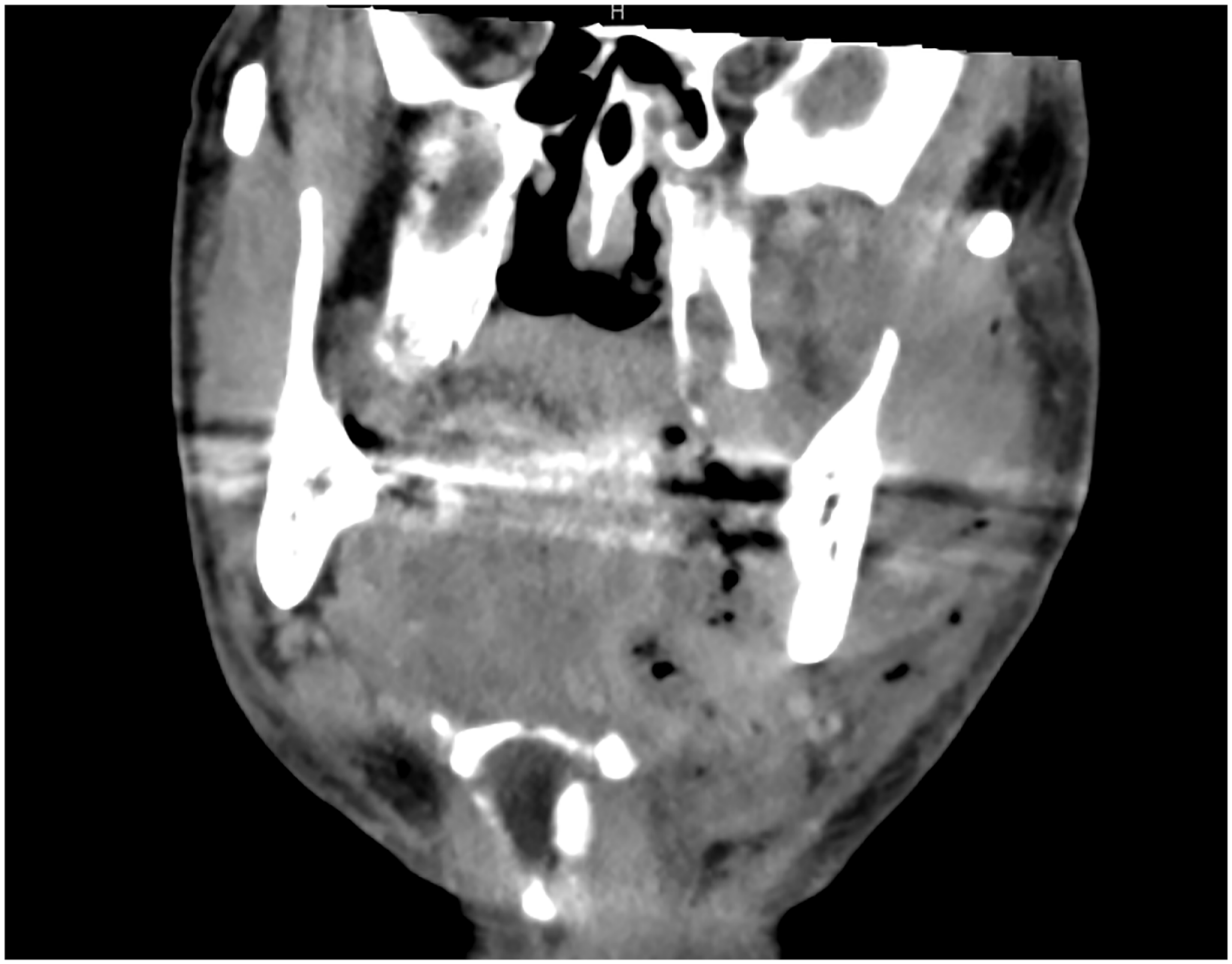
Contrast-enhanced CT scan demonstrating hallmark features of cervicofacial necrotizing fasciitis. Coronal contrast-enhanced computed tomography (CT) image of a patient with cervicofacial necrotizing fasciitis, illustrating characteristic radiologic features including diffuse fascial thickening, gas formation in the subcutaneous and deep cervical spaces, and fluid collections extending along fascial planes.

Early diagnosis is key to reducing mortality. Clinical suspicion should be confirmed with contrast-enhanced computed tomography (CT), which typically reveals fascial thickening, gas formation, and fluid collections along fascial planes [12, 13] (Figures 1 and 2). In some cases, magnetic resonance imaging (MRI) may offer higher soft tissue resolution, but its availability and speed are limiting factors in the acute setting [14]. Laboratory findings typically include elevated white blood cell counts, C-reactive protein (CRP), creatine kinase (CK), and lactate levels [15]. The Laboratory Risk Indicator for Necrotizing Fasciitis (LRINEC) score has been proposed as a diagnostic aid but has limited sensitivity in cervicofacial cases [16]. Several factors contribute to the reduced diagnostic utility of LRINEC in the head and neck region. First, gas-forming organisms, such as *Clostridium* spp., are less commonly involved in CNF than in infections of the trunk or limbs, which lowers the incidence of radiologically visible emphysema and correlating inflammatory patterns. Second, smaller anatomical compartments in the head and neck region may limit the extent of tissue destruction and systemic inflammatory response, particularly in the early stages—resulting in lower CRP, WBC, or creatinine values and thus underestimating LRINEC scores.

Moreover, overlapping laboratory profiles with deep neck abscesses, peritonsillar infections, or even severe pharyngitis can further reduce specificity. Thus, LRINEC should not be solely relied upon in the evaluation of suspected cervicofacial necrotizing infections. Instead, clinical vigilance and early imaging (CT or MRI) remain the cornerstone of timely diagnosis in locating the CNF in the head and neck region. Microbiological cultures from blood and wound samples often yield polymicrobial growth, including aerobic and anaerobic organisms. Group A Streptococcus (GAS) and Staphylococcus aureus (including MRSA) are common pathogens [17]. However, odontogenic NF is frequently polymicrobial with contributions from oral anaerobes such as Prevotella and Fusobacterium species [17, 18].

## DIFFERENTIAL DIAGNOSIS AND CLINICAL DISTINCTION

In its early stages, cervicofacial necrotizing fasciitis (CNF) may be difficult to distinguish from other deep neck infections. Common differential diagnoses include cellulitis, peritonsillar or parapharyngeal abscesses, Ludwig’s angina, and infected lymphadenopathy [17, 18]. These entities often present with swelling, erythema, and pain, but lack the rapid progression, skin changes (e.g., bullae, necrosis), crepitus, or systemic toxicity seen in NF [9–11]. Imaging plays a crucial role in differentiation. While cellulitis typically shows soft tissue edema without gas formation or fascial plane involvement, necrotizing fasciitis is characterized by subcutaneous emphysema, fluid tracking along fascial planes, and lack of tissue enhancement. A high index of suspicion, combined with early CT evaluation, remains critical for distinguishing NF from its mimics.

## MANAGEMENT

The cornerstone of NF treatment is early and aggressive surgical debridement, combined with broad-spectrum intravenous antibiotics and intensive supportive care [19, 20]. Table 1 summarizes the key diagnostic tools and therapeutic approaches currently recommended for cervicofacial NF (Table 1). Empiric antibiotic regimens should cover gram-positive, gram-negative, and anaerobic organisms, often including a combination of piperacillin/tazobactam plus clindamycin, as well as carbapenems or third-generation cephalosporins plus Metronidazole [19]. Antimicrobial regimens are later adjusted based on culture sensitivities. In many cases, repeated debridement is necessary within 24–48 hours [20, 21]. The surgical approach must be extensive and not limited to visible necrosis, often requiring dissection along fascial planes and removal of all non-viable tissue. Cervical incisions, mandibular release, and drainage of mediastinal extensions (if present) may be required [6]. Airway management is central to therapy [6, 22]. Elective tracheostomy is preferred in many cases due to anticipated prolonged airway compromise and the need for repeated surgeries [6]. Involvement of anesthesiologists and intensive care teams is essential early on. Hyperbaric oxygen therapy (HBOT) has been proposed as an adjunct treatment, particularly in cases of anaerobic infection and extensive tissue involvement, though high-level evidence for improved outcomes is limited [23]. However, the use of HBOT in NF remains controversial. While the theoretical benefits include enhanced oxygenation of hypoxic tissues, suppression of anaerobic bacteria, and improved leukocyte function, the clinical evidence is inconsistent, particularly in cervicofacial cases. A recent systematic review by Huang et al. concluded that while HBOT may reduce mortality and the number of debridements in selected cases, the heterogeneity of study designs, small sample sizes, and absence of randomized trials limit the strength of these conclusions [23]. Moreover, most available data pertain to extremity or truncal infections, with limited generalizability to head and neck NF.

**Table 1:** Diagnostic and therapeutic overview in cervicofacial necrotizing fasciitis

Aspect	Modality/Approach	Remarks
Surgical Timing	Immediate radical debridement	Key prognostic determinant
Empiric Antibiotic Therapy	Piperacillin/tazobactam + clindamycin; imipenem/meropenem/ertapenem + clindamycin; ceftriaxone + metronidazole + clindamycin	Broad-spectrum coverage including anaerobes
	Adjusted per culture sensitivity	Narrowed once pathogen(s) identified
Initial Imaging	Contrast-enhanced computed tomography (CT)	Rapid, widely available; detects gas, fascial thickening, fluid collections
	Magnetic resonance imaging (MRI)	Superior soft tissue contrast, but slower and less accessible in emergencies
Laboratory Tools	CRP, WBC, CK, Lactate	Elevated in most cases, but nonspecific
	LRINEC Score	Limited sensitivity in head and neck infections
Adjunctive Measures	Hyperbaric oxygen therapy (HBOT)	Controversial; may aid tissue salvage in selected cases

A recent systematic review by Tseros et al. (2023) analyzed 161 published cases of cervical necrotizing fasciitis (CNF) treated with HBOT and found a mortality rate of 7.6%, which is significantly lower than the 13.4% reported in an earlier review of CNF cases irrespective of HBOT use [24]. The authors concluded that HBOT may reduce mortality and complications in selected patients. However, the study also highlighted substantial limitations, including the absence of randomized controlled trials, heterogeneity of diagnostic criteria, small patient numbers, and a potential publication bias favoring positive outcomes. Moreover, most deaths occurred in patients with purely aerobic infections, suggesting that HBOT may be more effective in infections involving anaerobes. From a practical standpoint, access to HBOT is often restricted to specialized centers, and delays associated with transfer may offset potential benefits. Adverse effects such as barotrauma, oxygen toxicity, and logistic barriers must also be considered. Given these constraints, routine use of HBOT in cervicofacial NF cannot currently be recommended, but may be considered on a case-by-case basis—particularly in extensive anaerobic infections and when early surgical and antibiotic management is ensured.

## OUTCOME AND FOLLOW UP

Despite advances in supportive care, mortality rates in cervicofacial NF remain between 10 % and 30% [25]. Factors associated with poor prognosis include delayed surgical intervention, age >60 years, diabetes, renal insufficiency, and septic shock on admission [26, 27]. Functional and aesthetic sequelae are common and require reconstructive planning after infection control [28]. Long-term follow-up focuses on wound healing, scar contracture management, and restoration of oral functions [6, 28]. Psychological support is often necessary due to the traumatic nature of the disease and potential disfigurement.

## IMPLICATIONS FOR ONCOLOGY PATIENTS AND FUTURE DIRECTIONS

While NF is classically associated with metabolic disorders such as diabetes or trauma, cancer patients constitute a particularly vulnerable population. Immunosuppression due to chemotherapy, neutropenia, and radiotherapy-induced soft tissue changes are well-documented risk factors for soft tissue infections and may facilitate the rapid progression of necrotizing infections. Furthermore, head and neck cancer patients often present with anatomical alterations, impaired wound healing, or colonization with resistant flora, all of which complicate diagnosis and treatment. In our clinical experience, oncologic patients with cervicofacial NF frequently present atypically, and symptoms may be misattributed to tumor progression or treatment side effects. This underscores the need for heightened clinical vigilance and low threshold for imaging in this subgroup.

Importantly, there is a paucity of data addressing the outcomes of NF in cancer patients. We propose that future multicenter studies stratify NF patients by oncologic status to determine differential prognostic factors and treatment responses. Additionally, the development of early warning systems or biomarkers tailored to the immunocompromised host may improve early recognition and intervention. Given these considerations, cervicofacial NF should be recognized not only as a surgical emergency but also as a potential complication in oncologic care. Enhanced awareness among head and neck oncologists, radiation therapists, and oral surgeons is vital to ensure prompt diagnosis and treatment in this high-risk population.

## EXPERT OPINION: UNRESOLVED CHALLENGES AND RESEARCH NEEDS

Despite improved understanding of NF, several clinical dilemmas remain unresolved, particularly in cervicofacial cases. Based on our experience and review of the literature, we identify three major areas of ongoing debate and future investigation:

1. Extent and Timing of Surgical Debridement: While early radical surgery is widely accepted as the cornerstone of NF management, the optimal extent of debridement remains controversial. Involvement of the face and neck raises questions of surgical morbidity, aesthetic reconstruction, and preservation of function. More granular criteria to guide surgical aggressiveness in these anatomically complex regions are needed.
2. Limitations of Diagnostic Scoring Tools: Tools such as the LRINEC score are poorly validated in cervicofacial NF. Overreliance on such tools may delay surgical consultation. Future research should aim to develop scoring systems tailored to head and neck anatomy and symptomatology, possibly incorporating radiologic and molecular markers.
3. Adjunctive Therapies: The role of hyperbaric oxygen therapy (HBOT) remains debated. While theoretically beneficial for anaerobic infections and tissue oxygenation, high-level evidence from prospective studies is lacking. Additionally, the accessibility of HBOT is variable, making it an impractical standard in many institutions.

## CONCLUSIONS

Necrotizing fasciitis of the head and neck is a rare but devastating condition. Its successful management hinges on early clinical suspicion, prompt imaging, aggressive surgical debridement, airway protection, and broad-spectrum antibiotics. A multidisciplinary approach involving oral and maxillofacial surgeons, intensivists, infectious disease specialists, and reconstructive surgeons is vital to optimizing patient outcomes. Due to its rarity, further multicenter studies and the establishment of clinical registries may help better define prognostic factors and therapeutic strategies in cervicofacial NF.
